# Bromelain Ameliorates Atherosclerosis by Activating the TFEB-Mediated Autophagy and Antioxidant Pathways

**DOI:** 10.3390/antiox12010072

**Published:** 2022-12-29

**Authors:** Chia-Hui Chen, Chien-Chung Hsia, Po-An Hu, Chung-Hsin Yeh, Chun-Tang Chen, Cheng-Liang Peng, Chih-Hsien Wang, Tzong-Shyuan Lee

**Affiliations:** 1 Graduate Institute and Department of Physiology, College of Medicine, National Taiwan University, Taipei 10051, Taiwan; 2 Department of Isotope Application, Institute of Nuclear Energy Research, Taoyuan 32546, Taiwan; 3 Cardiovascular Surgery, Department of Surgery, National Taiwan University Hospital and College of Medicine, Taipei 10051, Taiwan

**Keywords:** bromelain, TFEB, AMPK, autophagy, reactive oxygen species, atherosclerosis

## Abstract

Bromelain, a cysteine protease found in pineapple, has beneficial effects in the treatment of inflammatory diseases; however, its effects in cardiovascular pathophysiology are not fully understood. We investigated the effect of bromelain on atherosclerosis and its regulatory mechanisms in hyperlipidemia and atheroprone apolipoprotein E-null (*apoe*^−/−^) mice. Bromelain was orally administered to 16-week-old male *apoe*^−/−^ mice for four weeks. Daily bromelain administration decreased hyperlipidemia and aortic inflammation, leading to atherosclerosis retardation in *apoe*^−/−^ mice. Moreover, hepatic lipid accumulation was decreased by the promotion of cholesteryl ester hydrolysis and autophagy through the AMP-activated protein kinase (AMPK)/transcription factor EB (TFEB)-mediated upregulation of autophagy- and antioxidant-related proteins. Moreover, bromelain decreased oxidative stress by increasing the antioxidant capacity and protein expression of antioxidant proteins while downregulating the protein expression of NADPH oxidases and decreasing the production of reactive oxygen species. Therefore, AMPK/TFEB signaling may be crucial in bromelain-mediated anti-hyperlipidemia, antioxidant, and anti-inflammatory effects, effecting the amelioration of atherosclerosis.

## 1. Introduction

Bromelain, a proteolytic enzyme, exists in the stem, fruit, leaves, and peel of pineapples. Stem bromelain has been revealed to possess several beneficial effects in humans and has been used for decades as a clinical medication to alleviate inflammation, coagulation disorders, and cancer [1,2,3]. Currently, it is used for the treatment of osteoarthritis and the prevention of swelling and inflammation after surgery [4,5]. In addition to the abovementioned benefits, emerging evidence indicates that bromelain also has beneficial effects on metabolic disorders [6,7,8]. Our previous study had indicated that bromelain upregulates the levels of proteins related to retinoid X receptor, peroxisome proliferator-activated receptor a (PPARα), and PPARγ, which are involved in the fatty acid β-oxidation for bioenergetics in mitochondria, leading to the decrease in high-fat diet (HFD)-induced lipid accumulation in the liver [7]. Recently, we further reported that bromelain alleviates NAFLD by activating the AMP-activated protein kinase (AMPK) autophagy signaling pathway [8]. Nevertheless, the effect of bromelain and the underlying mechanisms on the pathophysiology of the cardiovascular system require further investigation. Therefore, studies delineating the effects and mechanisms of bromelain in the development of cardiovascular diseases (CVDs) are warranted.

The liver is the most important organ in controlling aspects of lipid metabolism, including cholesterol and triglycerides, by regulating the lipid fluxes between the de novo lipid synthesis of liver, dietary, circulating, and peripheral local lipid pools [7,9,10]. To maintain the appropriate circulating levels of cholesterol, the delivery of apoB-containing lipoproteins from the liver to peripheral tissues, and high-density lipoprotein (HDL)-mediated reverse cholesterol transport from tissues back to the liver, are key regulatory mechanisms for the human body [11,12]. Either increases in the circulating level of low-density lipoprotein (LDL) or decreases in HDL-mediated cholesterol transport may result in hyperlipidemia, which is known to be the most important risk factor for the development of atherosclerosis. Atherosclerosis, a lipid deregulation and chronic inflammatory vascular disease, is characterized by excess lipid accumulation and persistent low-grade inflammation within the vessel wall [13,14,15,16], eventually resulting in severe clinical complications of arterial diseases, such as myocardial infarction and stroke [17,18,19,20]. On the other hand, the transcription factor EB (TFEB) plays a crucial role in regulating gene expression related to autophagy, lysosomal biogenesis, and antioxidants [21,22,23]. Recently, a growing body of studies have indicated that TFEB regulates the homeostasis of the cardiovascular system and confers protection from CVDs such as aortic aneurysms, cardiotoxicity, and atherosclerosis [24,25,26]. Moreover, TFEB decreases the inflammatory response and ROS production in endothelial cells and promotes autophagy and lysosomal biogenesis in macrophages, and it additionally leads to the alleviation of atherosclerotic progression [26,27]. These lines of evidence have emphasized the benefits of TFEB for vascular diseases; however, whether TFEB is involved in the bromelain-mediated protection from hepatic lipid accumulation and atherosclerosis requires further investigation.

In view of the effect of bromelain in the dysfunction of hepatic lipid metabolism, we aimed to investigate the effects and involved mechanisms of bromelain in the pathogenesis of atherosclerosis. First, we examined the effect of bromelain on adiposity, blood pressure, and the lipid profile by using a hyperlipidemia and athero-prone mouse model. Second, we investigated the effect of bromelain on hepatic lipid accumulation and atherosclerosis progression in *apoe*^−/−^ mice. Third, we delineated the involvement of the AMPK/TFEB signaling pathway in bromelain-conferred protection from hepatic lipid accumulation and atherosclerosis in *apoe*^−/−^ mice. Here, we provide new evidence to define the protective effect and molecular mechanism of bromelain on hepatic lipid metabolism and atherosclerosis.

## 2. Materials and Methods

### 2.1. Reagents

Bromelain was purchased from Cayman Chemical (Ann Arbor, MI, USA). Rabbit antibodies against inducible nitric oxide synthase (iNOS) were purchased from Santa Cruz Biotechnology (Santa Cruz, CA, USA). Rabbit antibodies against intercellular adhesion molecule 1 (ICAM-1), vascular cell adhesion molecule 1 (VCAM-1), C-X-C motif chemokine receptor 4 (CXCR4), NADPH oxidase 2 (NOX2), superoxide dismutase 1 (SOD1) and SOD2, and rat antibodies against F4/80 were obtained from Abcam (Cambridge, MA, USA). Rabbit antibodies for NOX1 and NOX4 were purchased from Proteintech (Rosemont, IL, USA). Mouse antibodies against 4-hydroxynonenal (4-HNE), goat antibodies against glutathione peroxidase (GPx), and cytokine ELISA kits were obtained from R&D Systems (Minneapolis, MN, USA). Rabbit antibodies against neutral cholesteryl ester hydrolase 1 (nCEH1) were obtained from Thermo Fisher Scientific Inc. (Waltham, MA, USA). Rabbit antibodies against heme oxygenase-1 (HO-1) were obtained from Assay Designs (Plymouth Meeting, PA, USA). Rabbit antibodies against LC3, sequestosome 1 (SQSTM1), and p-AMPK were purchased from Cell Signaling Technology (Danvers, MA, USA). Rabbit antibodies for lysosomal acid lipase (LAL) were obtained from GeneTex (Irvine, CA, USA). Rabbit antibodies for AMPK and mouse antibodies against β-actin were purchased from ABclonal (Woburn, MA, USA). Cholesterol, HDL-cholesterol (HDL-c), and triglyceride assay kits were purchased from Randox (Crumlin, Antrim, UK). The malondialdehyde (MDA) assay kit was obtained from Sigma-Aldrich (St. Louis, MO, USA). Total cholesterol, free cholesterol, cholesteryl ester, triglyceride, fatty acid, glycerol, and total antioxidant capacity fluorometric assay kits were obtained from BioVision (Milpitas, CA, USA).

### 2.2. Mice

This study conformed to the Guide for the Care and Use of Laboratory Animals (Institute of Laboratory Animal Resources, eighth edition, 2011). All animal experiments were approved by the Institutional Animal Care and Use Committee of the National Yang-Ming University (No. 1070314). *Apoe*^−/−^ mice were purchased from Jackson Laboratory (Bar Harbor, ME, USA). Mice were housed in barrier facilities and maintained under a 12 h/12 h light/dark cycle. The temperature (22 °C) and humidity (40–60%) of the vivarium were tightly controlled. Mice were housed in groups of 3–4 per cage and fed a regular chow diet containing 4.5% fat by weight (0.02% cholesterol) (Newco Distributors, Redwood, CA, USA). The *apoe*^−/−^ mice, at the age of 16 weeks, received oral administration of bromelain (20 mg/kg/day, n = 8) or phosphate-buffered saline (PBS; vehicle control, n = 8) for four weeks. The body weight; the weight of white adipose tissue (WAT), brown adipose tissue (BAT), and the liver; and the mean arterial pressure (MAP) of mice were measured. The mice were then euthanized with CO_2_ at the age of 20 weeks. At the end of the treatment, the plasma, heart, aorta, WAT, BAT, and the liver were collected and subjected to further experiments.

### 2.3. Histological Examination

The histological examination of the heart, WAT, BAT, and liver was performed as previously described [8,9]. The heart, WAT, BAT, and liver harvested from mice were fixed with 4% paraformaldehyde and serially dehydrated using increasing concentrations of alcohol (30%, 50%, 70%, 90%, and 100%), followed by treatment with xylene, for 60 min. The tissues were immersed in paraffin overnight at 60 °C and then embedded in paraffin. The tissue blocks were sectioned at 8 µm thickness. For histological examination, the sections of heart, WAT, BAT, and liver were subjected to hematoxylin and eosin (H&E) staining. For the quantification of atherosclerotic lesions, 50 serial sections from the aortic sinus of each mouse were collected. A total of 10–12 sections, sampled from every three consecutive sections, were deparaffinized and subjected to H&E staining. Photomicrographs of atherosclerotic lesions in the aortic sinus were obtained using a Motic TYPE 102M microscope (Motic Images Plus 2.0, Xiamen, China).

### 2.4. Plasma Lipid Profile Analysis

The plasma levels of cholesterol, HDL-c, and triglycerides were determined as previously described [8,9,10]. Blood was collected via a cardiac puncture. Plasma was isolated, and the levels of cholesterol, HDL-c, and triglycerides in plasma were determined using Spotchem EZ SP 4430 (ARKRAY, Kyoto, Japan).

### 2.5. Measurement of Inflammatory Cytokines

The concentrations of pro-inflammatory cytokines, including tumor necrosis factor-α (TNF-α), interleukin-1β (IL-1β), interleukin-6 (IL-6), monocyte chemoattractant protein-1 (MCP-1), and macrophage inflammatory protein 2 (MIP-2), in aortas were assessed according to the manufacturer’s instructions (R&D Systems, Minneapolis, MN, USA).

### 2.6. Western Blot Analysis

The expression of target proteins in the aortas and livers were evaluated as previously described [7,8,9,10]. Aortas and livers were lysed in a lysis buffer (50 mmol/L Tris pH 7.5, 5 mmol/L EDTA, 300 mmol/L NaCl, 1% Triton X-100, 1 mmol/L phenylmethylsulfonyl fluoride, 10 µg/mL leupeptin, and 10 µg/mL aprotinin), and proteins were separated electrophoretically on 8–15% sodium dodecyl sulfate–polyacrylamide gel. The proteins were then transferred onto a polyvinylidene fluoride (PVDF) membrane and blocked in 5% skim milk for 1 h at 37 °C. The blots were incubated with various specific primary antibodies, followed by incubation with horseradish peroxidase (HRP)-conjugated secondary antibodies. The protein bands were visualized using an enzyme-linked chemiluminescence detection kit (PerkinElmer, Waltham, MA, USA), and the band density was evaluated using TotalLab 1D (TotalLab, Newcastle Upon Tyne, UK). To ensure equal loading, β-actin was used as the internal control in each experiment.

### 2.7. Immunohistochemistry

The immunohistochemistry was performed as previously described [19]. The deparaffinized sections were incubated with a retrieval buffer for 10 min at 37 °C. The sections were then blocked with 2% BSA for 1 h at 37 °C, incubated with primary antibody overnight at 4 °C and then with FITC-conjugated or HRP- and FITC-conjugated secondary antibody overnight at 4 °C. Antigenic sites were visualized by adding 3,3-diaminobenzidine and observed under a Leica DMIRB microscope (Leica, Wetzlar, Germany) or a Nikon TE2000-U microscope (Nikon, Tokyo, Japan) with an image analysis system (QCapture Pro 6.0, QImaging, BC, Canada).

### 2.8. PET/CT Acquisition

PET/CT imaging was performed on a Bioscan scanner (Washington DC, USA) following an intravenous tail vein injection of [68Ga]-APD into *apoe*^−/−^ mice, as previously described [28]. PET/CT imaging was performed on a Bioscan scanner (matrix size, 128 × 128 × 159; CT attenuation-corrected; non-scatter-corrected) (Washington DC, USA) following an intravenous tail vein injection of approximately 14.8 MBq (0.4 mCi) of [68Ga]-APD into *apoe*^−/−^ mice (3-5 miceper group), as previously described [28]. Dynamic scans were acquired in a list mode format for at least 120 min and sorted into 22 frames, with 0.5 mm sinogram binsfor image reconstruction (4 × 15 s, 4 × 60 s, 11 × 300 s, 3 × 600 s). Mice were anesthetized with isoflurane (3% for induction and 2% for maintenance) throughout the experiment. *Apoe*^−/−^ mice were imaged with [68Ga]-APD (11.1 MBq) on treatment with or without bromelain to characterize differences in the severity of atherosclerotic lesions. To estimate the radioactivity concentration, volumes of interest were defined on coregistered PET/CT images using PMOD software. The target/background ratio (TBR) was calculated by placing a circular 2 mm volume of all studied organs/tissues around a site. TBRs were then calculated as the focal uptake divided by the blood pool.

### 2.9. Determination of Hepatic Lipids 

The levels of total cholesterol, free cholesterol, cholesteryl ester, triglycerides, fatty acids, and glycerol were evaluated as previously described [7,9,10]. They were evaluated using fluorescence assay kits (BioVision, Milpitas, CA, USA) according to the manufacturer’s instructions.

### 2.10. Lipid Peroxidation Assay 

The levels of MDA, a product of lipid peroxidation, in the aortas of bromelain-treated *apoe*^−/−^ mice were determined by the relevant assay kit (Sigma-Aldrich, St. Louis, MO, USA) according to the manufacturer’s instructions. Consequently, the results served as a biomarker for oxidative stress.

### 2.11. Total Antioxidant Capacity Assay

The levels of total antioxidant capacity, antioxidant proteins, and small molecules in the livers and aortas of bromelain-treated *apoe*^−/−^ mice were evaluated using fluorescence assay kits (BioVision, Milpitas, CA, USA) according to the manufacturer’s instructions.

### 2.12. Statistical Analysis

Comparisons between two groups were analyzed by the Mann–Whitney *U* test. SPSS v20.0 (SPSS Inc., Chicago, IL, USA) was used for statistical analysis. Differences were considered statistically significant at *p* < 0.05.

## 3. Results

### 3.1. Effect of Bromelain on Body Weight, Fat Tissue Weight, and Hyperlipidemia in apoe^−/−^ Mice

**Figure 1:**
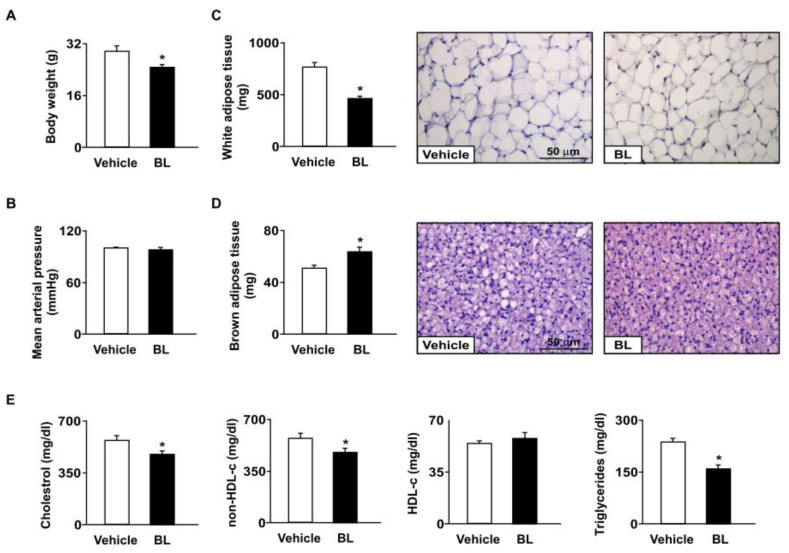
Effects of bromelain on body weight, blood pressure, adiposity, and hyperlipidemia in *apoe*^−/−^ mice. Male *apoe*^−/−^ mice were orally administered bromelain (BL, 20 mg/kg/day) or vehicle (PBS) for four weeks. (**A**) Body weight. (**B**) Mean arterial pressure. (**C**,**D**) The weight and histology of white adipose tissue and brown adipose tissue. Scale bar = 50 μm. (**E**) Plasma levels of total cholesterol, non-high-density lipoprotein cholesterol (non-HDL-c), HDL cholesterol (HDL-c), and triglycerides. * *p* < 0.05 vs. vehicle-treated *apoe*^−/−^ mice.

The *apoe*^−/−^ mice were used as our animal model. We first investigated the effects of bromelain on body weight, MAP, and adiposity in *apoe*^−/−^ mice. Daily treatment with bromelain for four weeks decreased the body weight without affecting MAP (Figure 1A,B). Additionally, bromelain decreased the weight of WAT; however, it increased BAT weight compared to that of vehicle-treated *apoe*^−/−^ mice (Figure 1C,D). The size of adipocytes in WAT (Figure 1C) and that of the lipid droplets (whitening) in BAT were decreased in bromelain-treated *apoe*^−/−^ mice (Figure 1D). Moreover, the plasma levels of total cholesterol, non-HDL-c, and triglycerides were significantly decreased in bromelain-treated *apoe*^−/−^ mice (Figure 1E). These findings suggest that bromelain confers a protective effect against adiposity and hyperlipidemia in *apoe*^−/−^ mice.

### 3.2. Bromelain Attenuates Aortic Inflammation and Results in the Retardation of Atherosclerosis in apoe^−/−^ Mice

**Figure 2:**
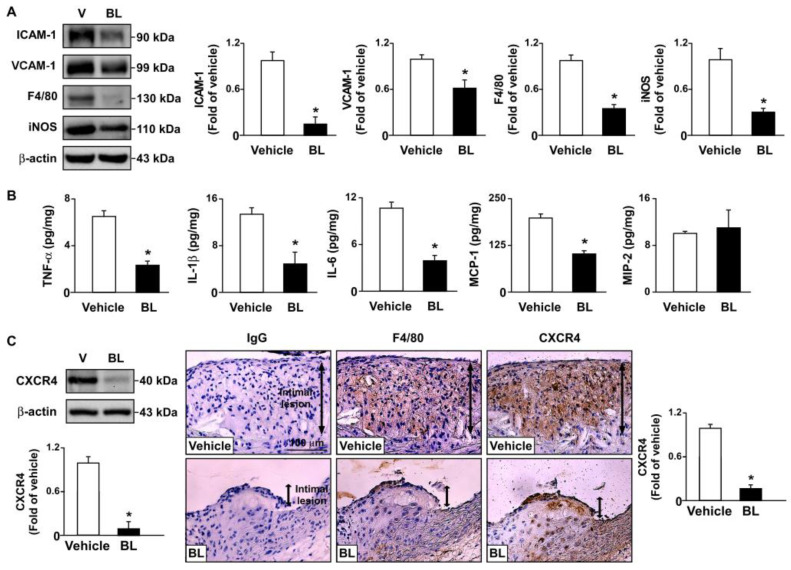
Effects of bromelain on aortic inflammatory response of *apoe*^−/−^ mice. Male *apoe*^−/−^ mice were orally administered bromelain (BL, 20 mg/kg/day) or vehicle (PBS) for four weeks. (**A**) Western blot analysis of ICAM-1 and VCAM-1, two endothelial dysfunction markers; F4/80, a macrophage marker; iNOS, an inflammation marker; and β-actin, in the atherosclerotic aortas of bromelain- or vehicle-treated *apoe*^−/−^ mice. (**B**) ELISA of TNF-α, IL-1β, IL-6, MCP-1, and MIP-2 in the aortas. (**C**) Western blot analysis of CXCR4 and β-actin in atherosclerotic aortas and immunohistochemistry for CXCR4, the most widely expressed chemokine receptor in physiological and pathological conditions. Cell nuclei were stained with hematoxylin. Scale bar = 100 µm. * *p* < 0.05 vs. vehicle-treated *apoe*^−/−^ mice.

**Figure 3:**
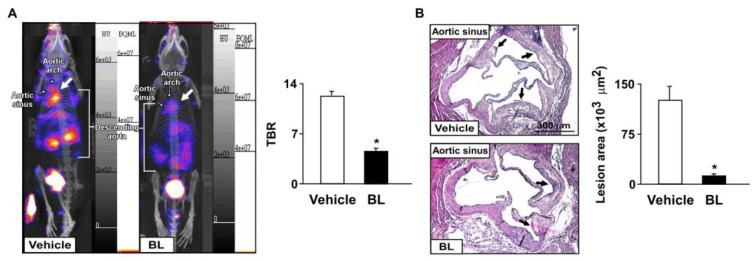
Bromelain attenuates atherosclerosis lesion size in *apoe*^−/−^ mice. Male *apoe*^−/−^ mice were orally administered bromelain (BL, 20 mg/kg/day) or vehicle (PBS) for four weeks. (**A**) The highest target/background ratio (TBR) of [68Ga]-APD on atherosclerotic aortas. (**B**) Atherosclerotic lesions at aortic roots, highlighted by H&E staining. Scale bar = 300 µm. Atherosclerotic lesions at the aortic sinus were indicated by arrows. * *p* < 0.05 vs. vehicle-treated *apoe*^−/−^ mice.

We then investigated the effects of bromelain on the inflammatory response and atherosclerosis progression. Our results showed that the daily administration of bromelain for four weeks significantly decreased the aortic levels of ICAM-1 and VCAM-1, F4/80, and iNOS in *apoe*^−/−^ mice (Figure 2A). Moreover, the levels of TNF-α, IL-1β, IL-6, MCP-1, and CXCR4, but not MIP-2, were decreased in the aortas of *apoe*^−/−^ mice (Figure 2B,C). These results suggest that bromelain inhibits the inflammatory response in *apoe*^−/−^ mice. We next used CXCR4-directed macrophage PET imaging with [68Ga]-APD to track the atherosclerotic lesions in *apoe*^−/−^ mice, as previously described [28,29,30,31]. The PET/CT images showed that treatment with bromelain decreased the TBR of [68Ga]-APD in *apoe*^−/−^ mice as compared to that of vehicle-treated *apoe*^−/−^ mice (Figure 3A). Histological examination with H&E staining showed that bromelain significantly decreased the sizes of the atherosclerotic lesions in the aortic sinuses of *apoe*^−/−^ mice (Figure 3B). These findings suggest that bromelain mitigates the inflammatory response and level of atherosclerosis in *apoe*^−/−^ mice.

### 3.3. Effects of Bromelain on Hepatic Levels of Lipids in apoe^−/−^ Mice

**Figure 4:**
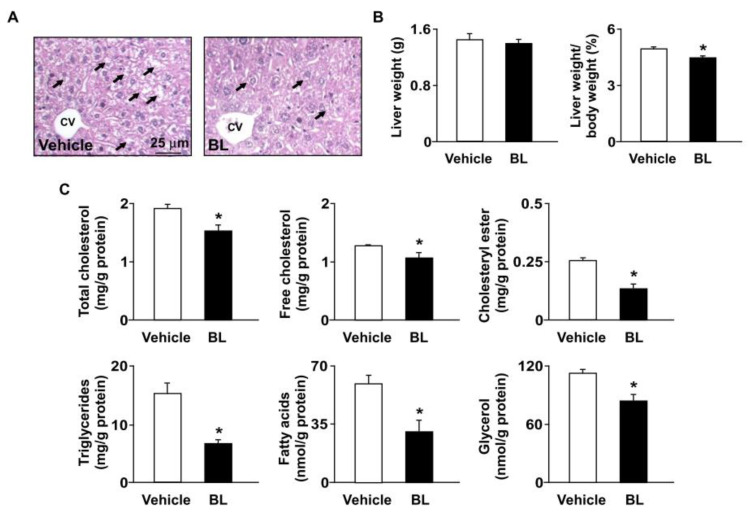
Bromelain reduces lipid accumulation in the livers of *apoe*^−/−^ mice. Male *apoe*^−/−^ mice were orally administered bromelain (BL, 20 mg/kg/day) or vehicle (PBS) for four weeks. (**A**) Representative images of H&E staining in bromelain- or vehicle-treated *apoe*^−/−^ mice. Scale bar = 25 μm. CV: central vein. The foamy hepatocytes were indicated by arrows. (**B**) The liver weight and the ratio of liver weight to body weight. (**C**) The hepatic levels of total cholesterol, free cholesterol, cholesteryl ester, triglycerides, fatty acids, and glycerol. * *p* < 0.05 vs. vehicle-treated *apoe*^−/−^ mice.

The liver plays a crucial role in regulating lipid metabolism [32]. We next investigated the effect of bromelain on the hepatic lipid metabolism of *apoe*^−/−^ mice. We found that treatment with bromelain for four weeks reduced lipid accumulation and the ratio of liver weight to body weight (Figure 4A,B). In addition, bromelain decreased the levels of total cholesterol, free cholesterol, cholesteryl ester, triglycerides, free fatty acids, and glycerol in the livers of *apoe*^−/−^ mice (Figure 4C). These findings indicate that bromelain has an advantageous effect on lipid metabolism in the livers of *apoe*^−/−^ mice.

### 3.4. TFEB Activation Is Involved in Bromelain-Induced Activation of Autophagy Flux and Antioxidant Capacity

**Figure 5:**
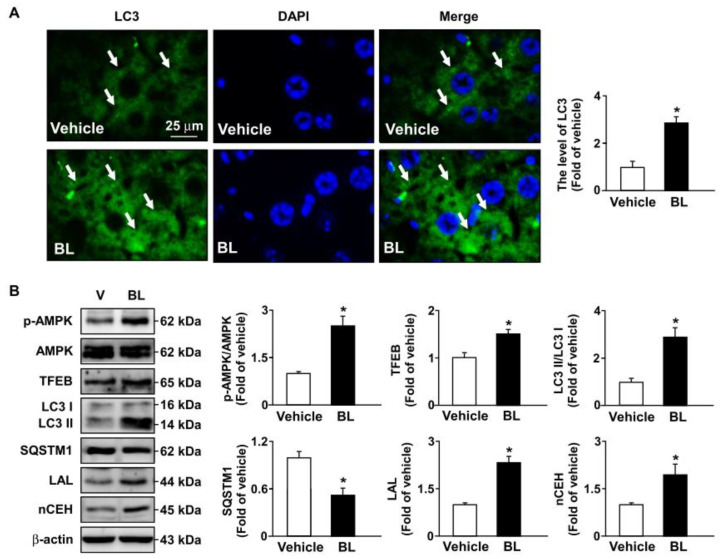
Bromelain triggers autophagy in the livers of *apoe*^−/−^ mice. Male *apoe*^−/−^ mice were orally administered bromelain (BL, 20 mg/kg/day) or vehicle (PBS) for four weeks. (**A**) Immunostaining with anti-LC3 antibody in the livers of bromelain- or vehicle-treated *apoe*^−/−^ mice. Scale bar = 25 μm. The hepatocytes with LC3 positive signals were indicated by arrows. (**B**) Western blot analysis of p-AMPK, AMPK, TFEB, LC3, SQSTM1, LAL, nCEH, and β-actin. * *p* < 0.05 vs. vehicle-treated *apoe*^−/−^ mice.

**Figure 6:**
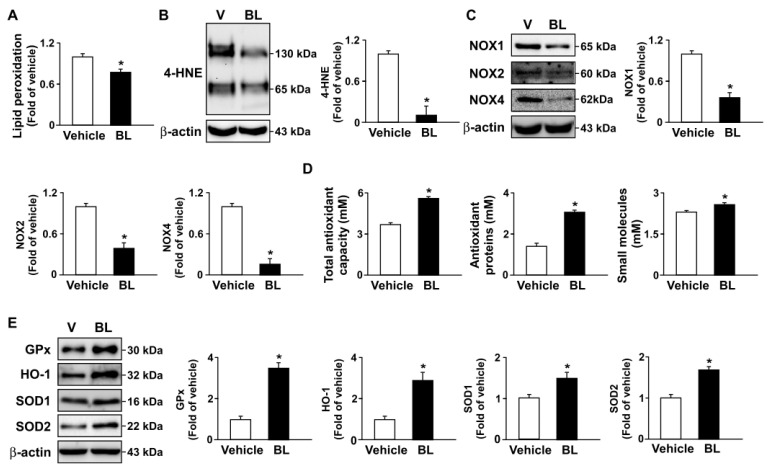
Bromelain decreases oxidative stress by increasing antioxidant capacity in the livers of *apoe*^−/−^ mice. Male *apoe*^−/−^ mice were orally administered bromelain (BL, 20 mg/kg/day) or vehicle (PBS) for four weeks. (**A**) The hepatic levels of lipid peroxidation in the livers of bromelain- or vehicle-treated *apoe*^−/−^ mice. (**B**,**C**) Western blot analysis of 4-HNE, NOX1, NOX2, NOX4, and β-actin. (**D**) The levels of total antioxidant capacity, antioxidant proteins, and small molecules. (**E**) Western blot analysis of GPx, HO-1, SOD1, SOD2, and β-actin. * *p* < 0.05 vs. vehicle-treated *apoe*^−/−^ mice.

The TFEB-mediated activation of autophagy is known to be a key event in orchestrating the metabolism of intracellular lipid droplets [33,34]. Therefore, we explored the role of the TFEB–autophagy pathway and found that bromelain conferred a beneficial effect on hepatic lipid accumulation. The results demonstrated that bromelain administration elicited the activation of the TFEB–autophagy pathway, as evidenced by the increase in the protein levels of LC3 and TFEB, and the decrease in the protein levels of SQSTM1, in the livers of *apoe*^−/−^ mice (Figure 5A,B). Additionally, we found that treatment with bromelain induced an increase in the phosphorylation of AMPK protein, which is an upstream regulator of the TFEB–autophagy pathway (Figure 5B). Moreover, bromelain upregulated the protein expression of LAL and nCEH, two key enzymes for the hydrolysis of lipids like triglycerides and cholesteryl ester (Figure 5B). These results suggest that bromelain may promote the hydrolysis of lipids by activating the AMPK–TFEB–autophagy pathway in the livers of *apoe*^−/−^ mice. On the other hand, TFEB has been reported to reduce ROS by upregulating antioxidant genes such as GPx, HO1, SOD1, and SOD2. We therefore examined whether the TFEB-mediated increase in antioxidant capacity is involved in the beneficial effects of bromelain on hepatic lipid accumulation in *apoe*^−/−^ mice. As shown in Figure 6, treatment with bromelain decreased the levels of lipid peroxidation and 4-HNE in the livers of *apoe*^−/−^ mice (Figure 6A,B). Moreover, bromelain downregulated the protein expression of NOX1, NOX2, and NOX4 in the livers of *apoe*^−/−^ mice (Figure 6C). Moreover, bromelain increased the antioxidant capacity, including the levels of total antioxidants, antioxidant proteins, and small molecules, as well as the protein levels of GPx, HO-1, SOD1, and SOD2, in the livers of bromelain-treated *apoe*^−/−^ mice (Figure 6D,E). These findings suggest that bromelain may increase antioxidant capacity by activating the AMPK–TFEB–autophagy pathway in the livers of *apoe*^−/−^ mice.

**Figure 7:**
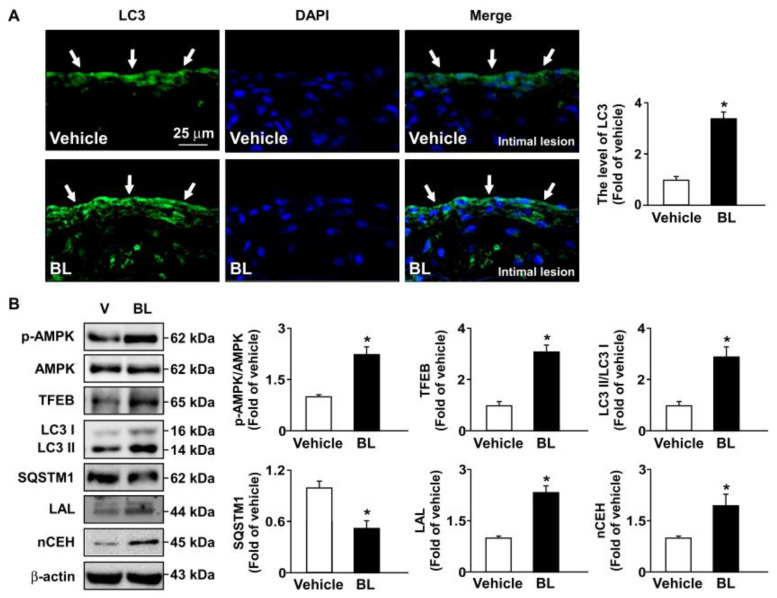
Bromelain activates autophagy in the aortas of *apoe*^−/−^ mice. Male *apoe*^−/−^ mice were orally administered bromelain (BL, 20 mg/kg/day) or vehicle (PBS) for four weeks. (**A**) Immunostaining with anti-LC3 antibody in aortas of bromelain- or vehicle-treated *apoe*^−/−^ mice. Scale bar = 25 μm. The vascular cells with LC3 positive signals were indicated by arrows. (**B**) Western blot analysis of p-AMPK, AMPK, TFEB, LC3, SQSTM1, LAL, nCEH, and β-actin. * *p* < 0.05 vs. vehicle-treated *apoe*^−/−^ mice.

**Figure 8:**
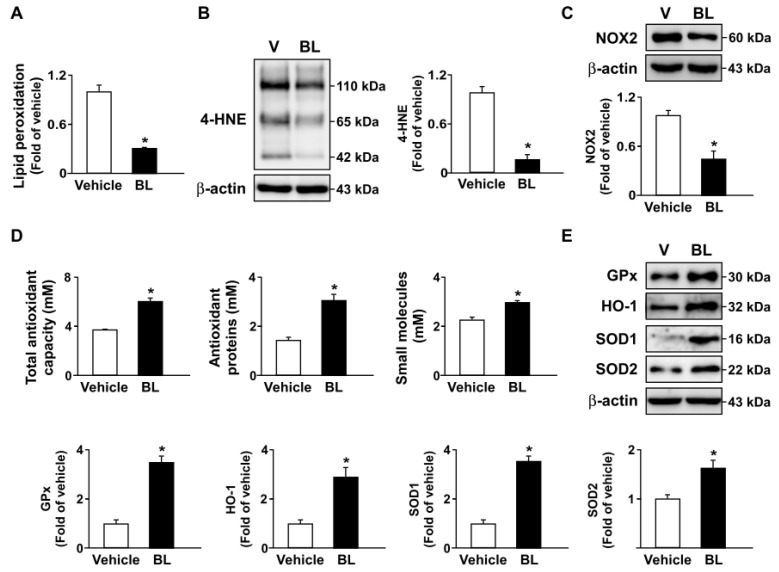
Bromelain decreases oxidative stress by increasing antioxidant capacity in the aortas of *apoe*^−/−^ mice. Male *apoe*^−/−^ mice were orally administered bromelain (BL, 20 mg/kg/day) or vehicle (PBS) for four weeks. (**A**) The aortic levels of lipid peroxidation of bromelain- or vehicle-treated *apoe*^−/−^ mice. (**B**,**C**) Western blot analysis of 4-HNE, NOX2, and β-actin. (**D**) The levels of total antioxidant capacity, antioxidant proteins, and small molecules. (**E**) Western blot analysis of GPx, HO-1, SOD1, SOD2, and β-actin. * *p* < 0.05 vs. vehicle-treated *apoe*^−/−^ mice.

**Figure 9:**
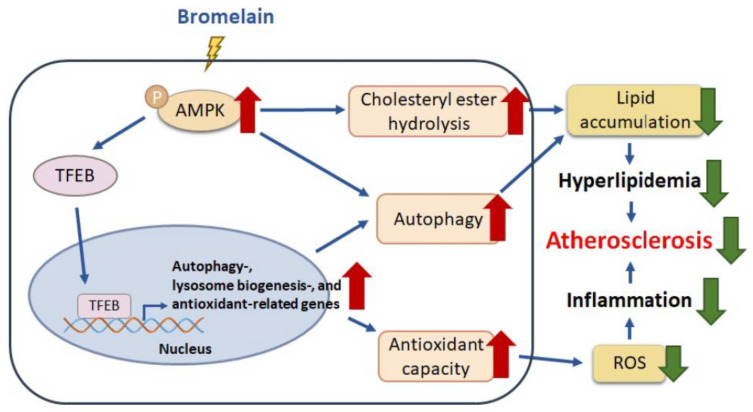
The proposed molecular mechanisms by which bromelain activates TFEB to reduce lipid accumulation, oxidative stress, and inflammation and also to retard atherosclerosis progression. Bromelain induces TFEB activation, which upregulates the expression of autophagy- and antioxidant-related proteins and alleviates lipid accumulation, oxidative stress and inflammation, leading to the retardation of atherosclerotic progression in *apoe*^−/−^ mice.

Moreover, similar results regarding the beneficial effects of bromelain on AMPK–TFEB pathway-mediated autophagy and antioxidant capacity were observed in the atherosclerotic aortas of *apoe*^−/−^ mice (Figure 7 and Figure 8). Together, these results indicate that the activation of the AMPK–TFEB pathway is important for the valuable effects of bromelain in regulating autophagy-mediated lipid hydrolysis and antioxidant capacity. This regulation leads to the alleviation of atherosclerosis in *apoe*^−/−^ mice (Figure 9).

## 4. Discussion

Bromelain is reported to exert protective effects against inflammatory diseases and NAFLD [1,2,3]. However, the effects of bromelain and the underlying molecular mechanisms in the physiology and pathology of the cardiovascular system remain elusive. We therefore used a hyperlipidemia- and atherosclerosis-prone mouse model to investigate the involved mechanism. In this study, we provide new evidence supporting the beneficial effect and regulatory mechanism of bromelain on the pathology of atherosclerosis. We found that treatment with bromelain for four weeks in *apoe*^−/−^ mice decreased the body weight and the organ weight, including of the WAT and the liver. These results were in line with the previous findings that the daily treatment of mice with bromelain for 12 weeks alleviated high-fat-diet-induced obesity as compared to vehicle-treated C57BL/6 mice [7]. Regarding the anti-obesity properties, Dave et al. reported that bromelain inhibits adipogenesis by inducing apoptosis and lipolysis in adipocytes [6]. We further demonstrated that bromelain improved hyperlipidemia and aortic inflammation in *apoe*^−/−^ mice and ultimately slowed down the progression of atherosclerosis. These findings suggest that bromelain has beneficial effects against atherosclerosis by regulating lipid metabolism and inflammation. However, the means by which bromelain regulates the hepatic lipid metabolism and aortic inflammatory response in *apoe*^−/−^ mice and its detailed molecular mechanism(s) remain blurred. Additional research exploring the cellular and molecular mechanisms underlying the anti-atherogenic action of bromelain is warranted.

Notably, Castell et al. reported that the pharmacokinetics of bromelain in human has been performed and that they found that, after oral administration with bromelain (3 g/day) for 3 days, the non-degraded bromelain can enter into circulation through intestinal absorption and retain its biological enzyme actimvity [35]. They demonstrated that the levels of bromelain in blood are range from 3.9 to 26.9 μg/mL after 600 mg oral administration in human. In this study, 20 mg/kg/day of bromelain was adopted. According to the Meeh–Rubner equation, the daily intake of 20 mg/kg of bromelain for mice is comparable to the daily intake of 200 mg of bromelain for humans [7]. Moreover, our preliminary results demonstrated that boiled bromelain failed to inhibit the oleic acid-induced lipid accumulation in hepatocytes (data not shown). Therefore, we thought that the proteolytic activity is required for the anti-atherogenic action of bromelain. Nevertheless, the molecular mechanism underlying the beneficial effect of bromelain and its target proteins on the enzymatic activity remains to be investigated.

In addition to pro-inflammatory cytokines such as TNF-α, IL-1β, IL-6, and MCP-1, chemokines such as macrophage migration inhibitory factor and CXCL12 play an important role in the initiation and the progression of arteriosclerosis [28,29,30,31]. CXCR4, the specific receptor for CXCL12, is expressed in the vascular cells and macrophages and plays a crucial role in leukocyte recruitment into vessel walls and monocyte–macrophage differentiation, as well as macrophage polarization, during the development of atherosclerosis [35,36]. CXCL12 promotes atherosclerosis by deregulating the cholesterol metabolism of macrophage foam cells and leads to the acceleration of atherosclerosis [37,38]. In contrast, a functional CXCR4 blockade exacerbates the progression of atherosclerosis [39], and the cell-specific deletion of CXCR4 in arterial endothelial cells or smooth muscle cells accelerates the progression of atherosclerosis in *apoe*^−/−^ mice [40]. These lines of evidence suggest that the role of CXCL12 and CXCR4 in atherosclerosis is still under debate. Nevertheless, our results showed that treatment with bromelain decreased the aortic levels of TNF-α, IL-1β, IL-6, MCP-1, and CXCR4, and consequently lessened the size of atherosclerotic lesions. This is in the agreement with the previous findings that the inhibition of the inflammatory response within the artery wall decelerates atherosclerotic progression [41,42]. Based upon our observations, we further confirmed the anti-atherogenic effect of bromelain by using PET/CT imaging with [68Ga]-APD, which has been designed as a PET tracer of CXCR4 for the imaging of atherosclerosis [43]. 

Autophagy is an indispensable biological process by which cytoplasmic components are sequestered in double-membrane vesicles and degraded on fusion with lysosomal compartments [33]. Hu et al. reported that bromelain triggers the activation of autophagy, which is closely regulated by AMPK and plays an important role in treating NAFLD and related lipid disorders [7,8]. Additionally, TFEB activity is also regulated by AMPK phosphorylation [27,44]. Under physiological conditions, TFEB is located in the cytoplasm; however, certain conditions, such as starvation, lysosomal dysfunction, or oxidative stress by lipid accumulation, induce the translocation of TFEB to the nucleus, where it stimulates the transcription of its target genes, including autophagic and lysosomal genes [45,46,47]. Our results further confirmed this notion. Treatment with bromelain induced the levels of LC3 puncta in the liver and atherosclerotic lesions of *apoe*^−/−^ mice and the phosphorylation of AMPK protein expression, leading to upregulated TFEB and triggering the expression of autophagy-related proteins, including LC3-I and LC3-II, but reducing the protein levels of SQSTM1, suggesting that AMPK phosphorylation is required for the activation of TFEB. Triggering the autophagy pathway leads to the beneficial effects of bromelain in reducing lipid metabolism, both in the liver and atherosclerotic lesions of *apoe*^−/−^ mice. These results are consistent with previous findings that fenofibrate, a PPARα agonist, improved hepatic lipid accumulation by upregulating TFEB-mediated lipophagy [48]. In view of its function, TFEB activation, induced by nuclear receptor PPARα with currently available drugs or new molecules, might be a therapeutic target for the treatment of metabolic diseases.

Oxidative stress is an imbalance between pro-oxidants and antioxidants, have often been inconsistent in demonstrating health benefits in terms of quantitative measures of disease outcome [49,50]. Furthermore, oxidative stress can be quantified in humans as the redox state of plasma glutathione/oxidized glutathione ratio (SH/GSSG) [50]. Several lines of evidence indicate that the deregulation of NOX–ROS signaling plays an important role in the key events in the development of atherosclerosis [51,52,53,54]. Targeting the ROS pathway with antioxidants has therapeutic value in preventing oxidative stress-mediated metabolic disorders [51,52,53,54]. As a consequence, antioxidant therapy is a typical method for atherosclerosis treatment. Thus, the TFEB-mediated increase in antioxidant capacity could partially explain its anti-inflammatory function [55]. Interestingly, our data revealed that treatment with bromelain decreased ROS generation and NOX activity in the livers and aortas of *apoe*^−/−^ mice. Moreover, the levels of antioxidants were significantly increased in the livers and aortas of bromelain-treated *apoe*^−/−^ mice. Our results suggest that TFEB activation is required to trigger the autophagy pathway under the beneficial effects of bromelain in reducing lipid metabolism in the aortas of *apoe*^−/−^ mice. Our findings are consistent with those of Lu et al. (2017), who found that TFEB overexpression in endothelial cells decreased the intracellular ROS and upregulated the gene expression of antioxidants HO-1 and SOD2, which was attributed to the anti-inflammatory effect of TFEB [56,57]. In view of its function, treatment with bromelain confers protection against oxidative stress and inflammatory responses, leading to the alleviation of the progression of atherosclerosis by activating TFEB to stimulate antioxidant generation.

## 5. Conclusions

In conclusion, our findings elucidate the novel function of bromelain. This suggests that TFEB activation to stimulate autophagy- and antioxidant-related proteins is the mechanism by which bromelain provides protection from the deregulation of lipid metabolism, oxidative stress, and inflammation, leading to the mitigation of atherosclerosis (Figure 9). Our study reveals a new molecular mechanism underlying the protective effects of bromelain on atherosclerosis. We thus offer supporting evidence for a link between bromelain and TFEB, a link which activates the autophagy component and antioxidant generation in the liver and aortas. Our findings are vital for a better understanding of the regulatory mechanism of bromelain and for the identification of new therapeutic targets for the treatment of atherosclerosis.

## Data Availability

The original contributions presented in the study are included in the article. Further inquiries can be directed to the corresponding author.
