# Correction: Chen et al. Bromelain Ameliorates Atherosclerosis by Activating the TFEB-Mediated Autophagy and Antioxidant Pathways. *Antioxidants* 2023, *12*, 72

**DOI:** 10.3390/antiox13070830

**Published:** 2024-07-11

**Authors:** Chia-Hui Chen, Chien-Chung Hsia, Po-An Hu, Chung-Hsin Yeh, Chun-Tang Chen, Cheng-Liang Peng, Chih-Hsien Wang, Tzong-Shyuan Lee

**Affiliations:** 1 Graduate Institute and Department of Physiology, College of Medicine, National Taiwan University, Taipei 10051, Taiwan; chiahuichen1993@ntu.edu.com (C.-H.C.); d07441003@ntu.edu.tw (P.-A.H.); 2 Department of Isotope Application, Institute of Nuclear Energy Research, Taoyuan 32546, Taiwan; hsiacc@iner.gov.tw (C.-C.H.); yehch@iner.gov.tw (C.-H.Y.); ctchen@iner.gov.tw (C.-T.C.); clpeng@iner.gov.tw (C.-L.P.); 3 Cardiovascular Surgery, Department of Surgery, National Taiwan University Hospital and College of Medicine, Taipei 10051, Taiwan

## Error in Figure

In the original publication [1], there was a mistake in Figure 8E as published. In Figure 8E, the images of GPx, HO-1, and β-actin were not correctly exchanged with those in Figure 6E. The corrected Figure 8 appears below. The authors state that the scientific conclusions are unaffected. This correction was approved by the Academic Editor. The original publication has also been updated. 
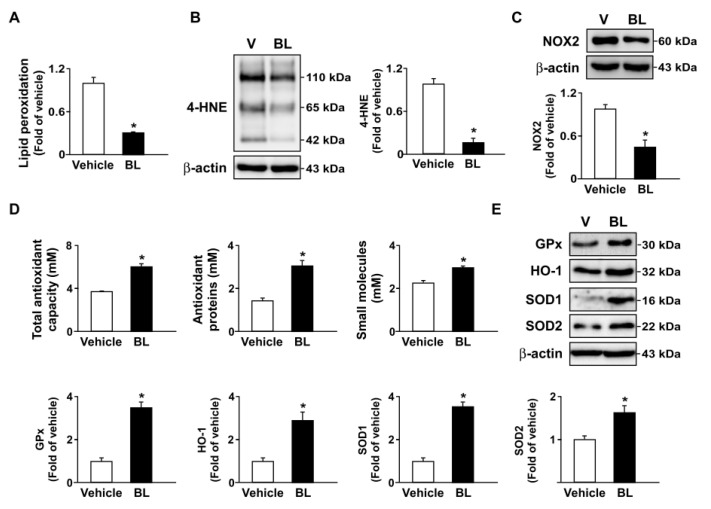


## References

[1] Chen C.-H., Hsia C.-C., Hu P.-A., Yeh C.-H., Chen C.-T., Peng C.-L., Wang C.-H., Lee T.-S. (2023). Bromelain Ameliorates Atherosclerosis by Activating the TFEB-Mediated Autophagy and Antioxidant Pathways. Antioxidants.

